# CVD graphene as an electrochemical sensing platform for simultaneous detection of biomolecules

**DOI:** 10.1038/s41598-017-07646-2

**Published:** 2017-08-01

**Authors:** Xiaodan Wang, Delan Gao, Mingji Li, Hongji Li, Cuiping Li, Xiaoguo Wu, Baohe Yang

**Affiliations:** 1 Tianjin Key Laboratory of Film Electronic and Communicate Devices, School of Electrical and Electronic Engineering, Tianjin University of Technology, Tianjin, 300384 P.R. China; 2 Tianjin Key Laboratory of Organic Solar Cells and Photochemical Conversion, School of Chemistry and Chemical Engineering, Tianjin University of Technology, Tianjin, 300384 P.R. China

## Abstract

The development of electrochemical biosensors for the simultaneous detection of ascorbic acid (AA), dopamine (DA), uric acid (UA), tryptophan (Trp), and nitrite (${{\rm{NO}}}_{2}^{-}$) in human serum is reported in this work. Free-standing graphene nanosheets were fabricated on Ta wire using the chemical vapor deposition (CVD) method. CVD graphene, which here served as a sensing platform, provided a highly sensitive and selective option, with detection limits of AA, DA, UA, Trp, and ${{\rm{NO}}}_{2}^{-}$ of 1.58, 0.06, 0.09, 0.10, and 6.45 μM (S/N = 3), respectively. The high selectivity of the electrode is here explained by a relationship between the bandgap energy of analyte and the Fermi level of graphene. The high sensitivity in the oxidation current was determined by analyzing the influence of the high surface area and chemical structure of free-standing graphene nanosheets on analyte adsorption capacity. This finding strongly indicates that the CVD graphene electrode can be used as a biosensor to detect five analytes in human serum.

## Introduction

Electrochemical biosensors that have high sensitivity have found widespread use in clinical diagnosis^1–4^. Human body fluids contain electrolytes, including a wide variety of electroactive materials, including biological, organic, and inorganic electrolytes. L-ascorbic acid (AA), dopamine (DA), uric acid (UA), tryptophan (Trp), and nitrite (${{\rm{NO}}}_{2}^{-}$) are found in body fluids. Scurvy is caused by a deficiency of AA^5^, and abnormal DA transmission has been implicated in Huntington’s disease and neuroendocrine disorders^6^. Abnormalities in UA levels indicate symptoms of gout, hyperuricemia, and leukemia^7, 8^; Trp is an essential amino acid and that is a serotonin precursor, Parkinson’s disease linked to Trp^9^; ${{\rm{NO}}}_{2}^{-}$ is an important precursor in the formation of N-nitrosamines, many of which have been shown to be potent carcinogens in humans^10–12^. AA, DA, UA, Trp, and ${{\rm{NO}}}_{2}^{-}$ usually coexist in body fluids^13, 14^. Thus, the development of sensors for the simultaneous detection of AA, DA, UA, Trp, and ${{\rm{NO}}}_{2}^{-}$ with sensitivity and selectivity is highly important to the investigation of their physiological functions and to diagnostic and analytical applications.

One major problem troubling the development of such sensors is that AA, DA, UA, Trp, and ${{\rm{NO}}}_{2}^{-}$ all undergo oxidation at the closed potentials of conventional electrodes. This mutual interference from each other should be expected and must be taken into account. In addition, conventional electrodes have a pronounced fouling effect, which can result in poor selectivity and reproducibility. To overcome these obstacles, so far, various materials have been used to modify conventional electrodes for the simultaneous detection of AA, DA, and UA^15–17^; of UA and Trp^18^; of Trp and ${{\rm{NO}}}_{2}^{-}$, of DA, UA and Trp^19, 20^; of AA, DA, UA, and Trp^21, 22^; and of AA, DA, UA, and ${{\rm{NO}}}_{2}^{-}$. Unfortunately, there have been no reports that the simultaneous determination of all five. Many reported methods of simultaneous detection (for two or three or four of these electroactive compounds) are complicated, expensive, unstable, and have sensing layers that detached from the basic electrode easily.

Graphene is one of the most commonly used materials in electrodes, in which it serves as a sensitive film. The graphene materials include graphene oxide (GO), reduced graphene oxide (rGO), and chemical vapor deposition (CVD) graphene. Of these, GO and rGO are the most commonly used in electrochemical sensors^23–27^. According to the references, the GO composite-modified-electrodes were detected simultaneously with the DA and 5-hydroxytryptamine^28^, DA and UA^29^, guanine and adenine^30^, AA, DA, and UA^31, 32^, AA, DA, UA, and folic acid^33^, and l-tyrosine and l-tyrosine^34^. However, these graphene-based materials use of drip coating, electrodeposition, and screen printing method, these graphene based-materials are modified to the basis electrodes. Weak adhesion between the sensitive film and the based electrode can shorten the life of the sensitive layer. They also require special storage conditions. CVD graphene has higher electrical conductivity and fewer defects than graphene prepared using wet-chemical methods^35^. CVD graphene is grown directly on the conductor, and the strong binding between the two prevents them separating from each other^36, 37^. These give the electrodes strong stability and long life. The CVD graphene-based sensor has been used to detect levodopa^38^ and glucose^39, 40^. Further increases in the surface area of graphene electrodes have been found to enhance the adsorption capacity of graphene layer for the analyte^41, 42^, thereby increasing the sensitivity of the graphene electrode. To achieve this, the vertical growth of graphene must be fostered to form a graphene wall on a metal substrate. Moreover, these graphene walls would ideally be highly dense, and the high edge density increases the charge storage ability^43^, thereby increasing adsorption and accelerating the electron transfer rate. More importantly, free-standing graphene walls are regularly exposed to different edge structures, which indicates the presence of different band-gap energy levels on graphene layer^44^. This renders the electronic transition process different from that of other graphene^45^, then the sensitive layer may have a better potential selectivity. Recently, a method of directly synthesizing graphene electrodes using CVD has been developed. This made the production of high-quality graphene with vertically oriented features possible^46^.

Based on these above considerations, a biosensing electrode with CVD graphene as sensitive layer was here developed, and Ta wire served as the basis electrode. The present work focuses on evaluating the simultaneous detection of AA, DA, UA, Trp, and ${{\rm{NO}}}_{2}^{-}$, and response mechanism of this graphene designed for the simultaneous detection of multiple analytes is evaluated.

## Results

### Characterization of graphene/Ta wire electrodes

**Figure 1:**
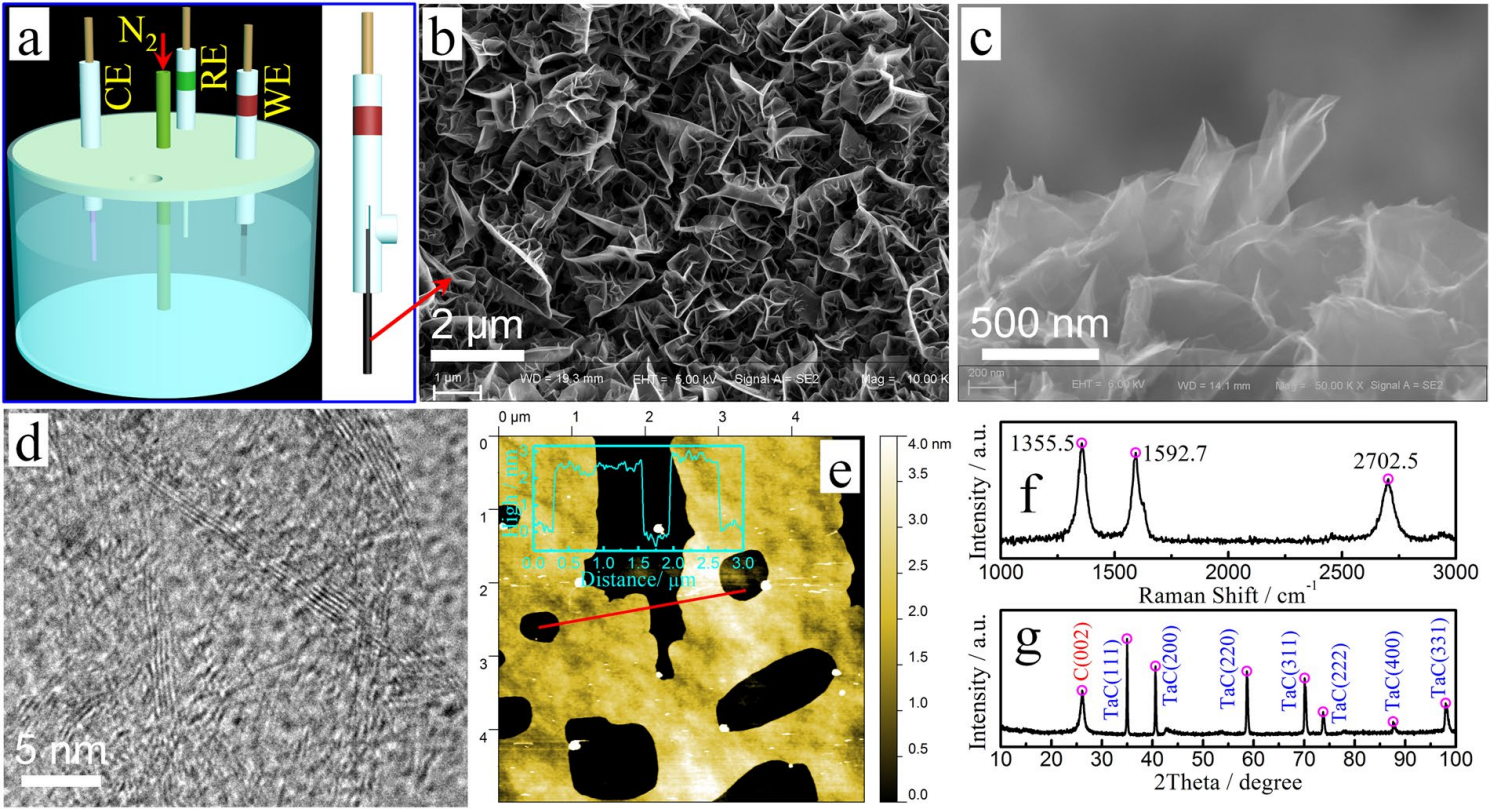
(**a**) Schematic diagram of electrolytic cell and electrodes. Dissolved oxygen was removed by passing N_2_ into the electrolytic cell. Counter electrode (CE), reference electrode (RE), and working electrode (WE) are platinum sheet, saturated calomel electrode, and graphene/Ta wire, respectively. (**b**) Top-view and (**c**) cross-sectional SEM images of graphene nanosheets. (**d**) High-resolution TEM image of graphene nanosheets. (**e**) AFM image of graphene nanosheets. (**e**) Inset showing height profile along line. (**f**) Raman spectrum and (**g**) XRD pattern of graphene/Ta wire.

Figure 1a shows the three-electrode testing system with the graphene/Ta wire as the sensing electrode. Figure 1b and c clearly indicate that graphene layers with pores between the graphene sheets formed an open network. The graphene sheets were thin and transparent, and the thinner sheet was divided from the surface of the nanosheet. The graphene layer contained many curved nanosheets with vertical growth trends. Network structures of graphene nanosheets provide more channels between the graphene sheets to facilitate the entrance of electrolyte ions or electrons into the inter-space. The effective area of graphene layers calculated using electrochemical kinetics is 2.24 cm^2^ (Fig. S1), which is ten times the actual area (0.2262 cm^2^) of graphene/Ta wire electrode. The thickness of graphene nanosheets can be confirmed using high-resolution transmission electron microscopy (TEM) (Fig. 1d) and atomic force microscopy (AFM) (Fig. 1e) images. The thickness of these graphene sheets ranged from 0.34 nm to 3 nm, corresponding to stacking of approximately 1–10 layers of the monoatomic graphene sheets. The conductivity of graphene affects the sensitivity of the sensing electrode. The conductivity of multi-layer graphene is lower than that of single-layer graphene. Compared with the low-layer graphene prepared by the liquid-phase oxidation method, the vertical multi-layer graphene sheets prepared using the CVD method have high orientation, low contact resistance (between the Ta wire and graphene layer), and less oxygen-containing groups, which impart high electrical properties to the prepared graphene/Ta wire. Of course, if we can prepare a graphene electrode with vertically arranged single-layer graphene sheets, then the sensitivity of the graphene electrode will be higher than the current graphene/Ta wire.

The Raman spectrum of graphene layer is shown in Fig. 1f. The spectrum feature include a D band at approximately 1355.5 cm^−1^, G band at around 1592.7 cm^−1^, and 2D band at approximately 2702.5 cm^−1^. The D band is a characteristic feature, indicating defects. It can be caused by excitation of the edges of graphene sheets and their random orientation^47, 48^. The G band shows sp^2^ vibrations in the graphitic plane, which confirms the presence of graphene sheets. Intense, well-defined 2D bands with a narrow width of full width at half maximum is peculiar for few layer graphene. The X-ray diffraction (XRD) pattern of the graphene/Ta wire is shown in Fig. 1g. A sharp peak at about 2θ = 26° corresponding to the (002) reflection of graphite (JCPDS 75–1621) was observed with interlay space (d-spacing) of 0.34 nm, indicating the graphene layer formed on Ta wire. In addition, the TaC phases were observed, which indicated that the carbon atoms first dissolved into the interior of Ta which formed the TaC transition layer and then formed graphene nanosheets through further deposition of carbon atoms^46^.

### Electrochemical behaviors of graphene electrode

**Figure 2:**
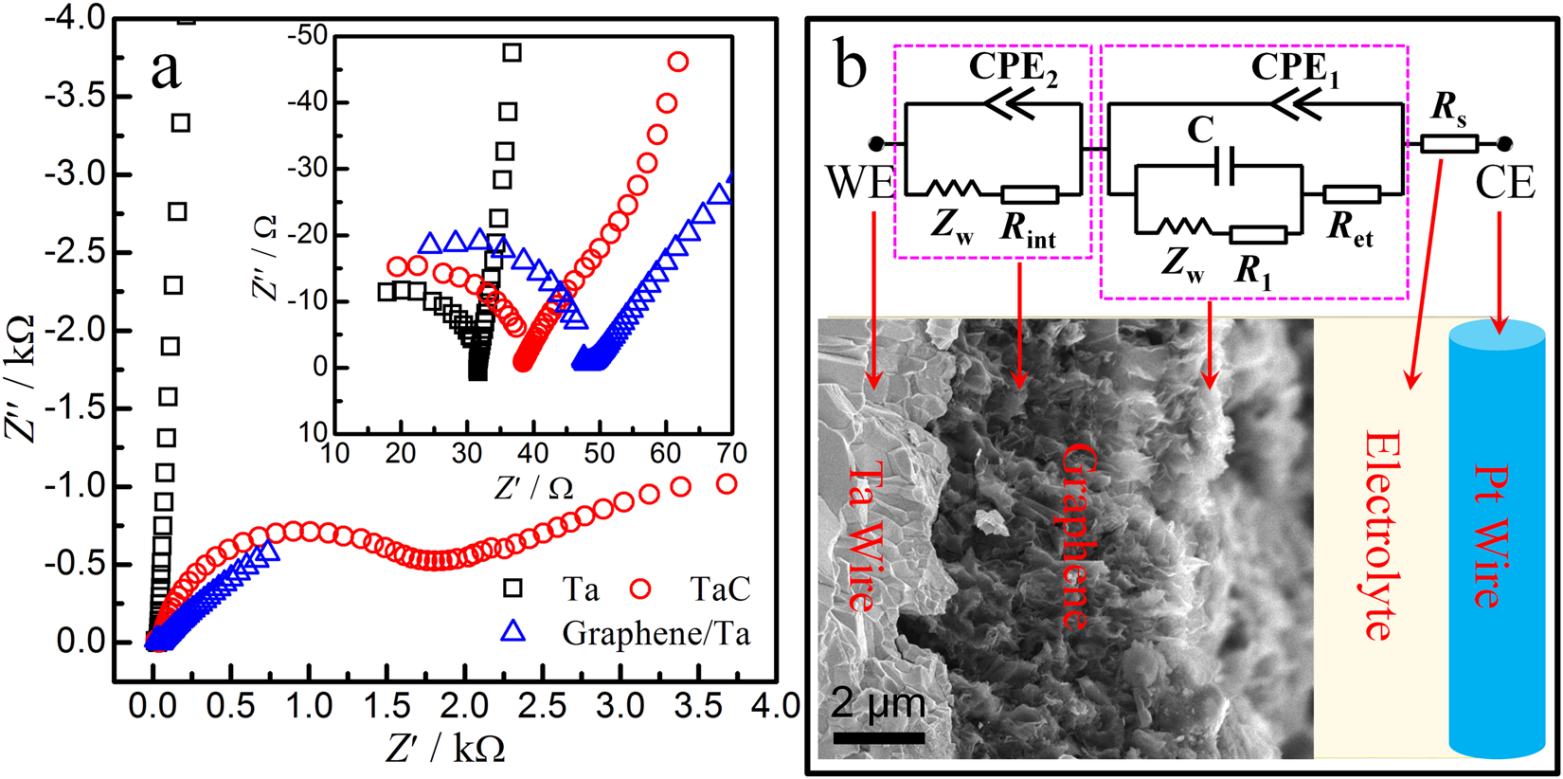
(**a**) Impedance spectra of the Ta wire, TaC/Ta wire, and graphene/Ta wires in 0.1 KCl containing 5 mM K_3_Fe(CN)_6_ and 5 mM K_4_Fe(CN)_6_.

Figure 2a compares the Nyquist plots of pure Ta wire, TaC/Ta wire, and graphene/Ta wire in 0.1 M KCl solution containing 5.0 mM K_3_[Fe(CN)_6_] and 5.0 mM K_4_[Fe(CN)_6_], respectively. For the TaC/Ta wire, the Nyquist plot consisted of two semicircles in high- and medium-frequency regions followed by an inclined line in low-frequency region. For the graphene/Ta wire, the Nyquist plots consisted of a semicircle in high-frequency region followed by an inclined line in a medium-low-frequency region. According to the Randles-type circuit in Fig. 2b, the series resistance (*R*_s_) is created by the electrolyte. The semicircle indicates the charge-transfer resistance (*R*_ct_) at the external graphene/electrolyte interface; the interface resistance (*R*_int_) is contributed from the bottom graphene layer. As shown, the diameter of the semicircle for the TaC/Ta wire (*R*_ct_ = 200 Ω cm^2^) is much larger than that of the graphene/Ta wire (*R*_ct_ = 37.2 Ω cm^2^), indicating the graphene/Ta wire electrode exhibited better charge transfer performance than the TaC/Ta wire and had excellent conductivity.

**Figure 3:**
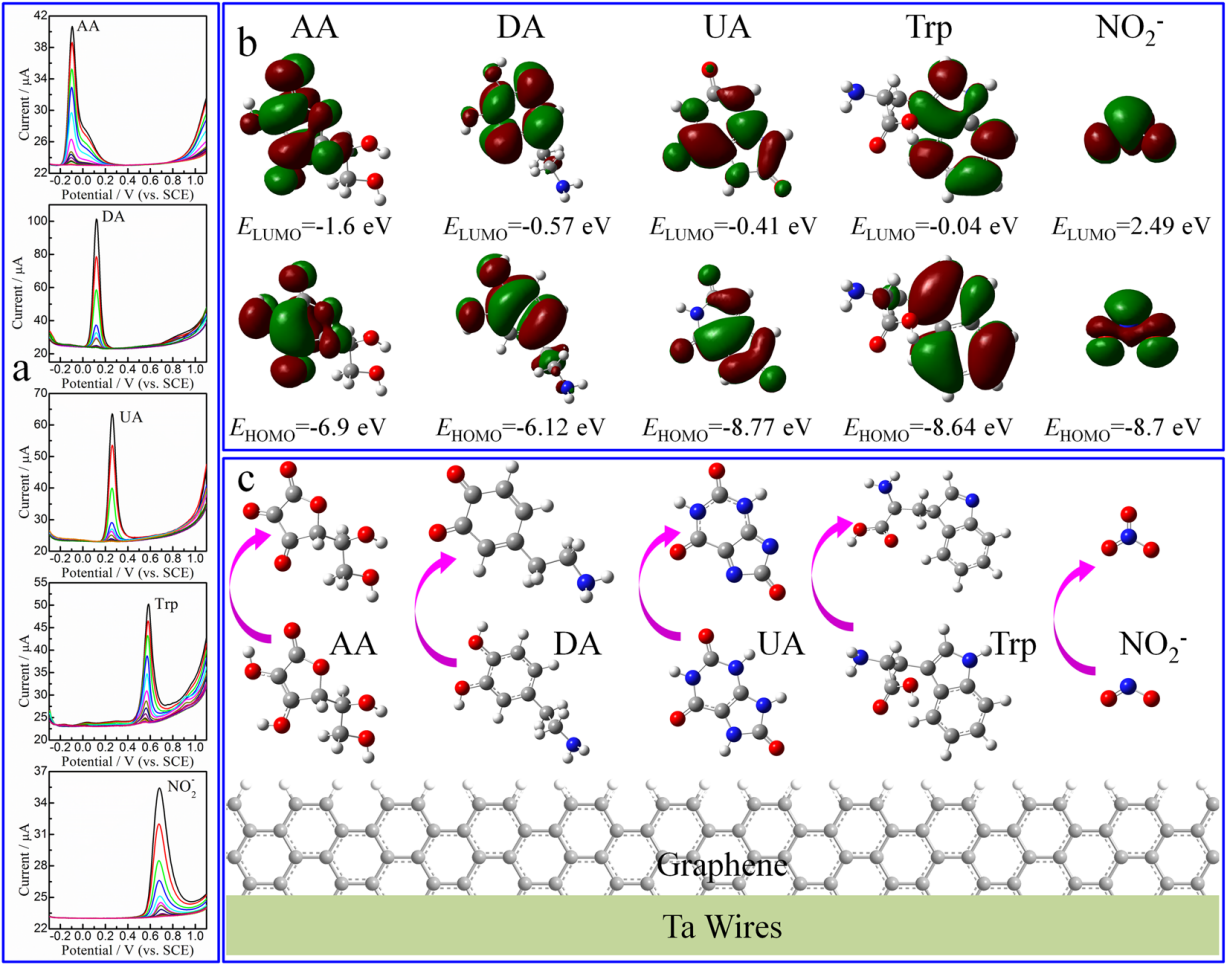
(**a**) DPVs of graphene/Ta wire electrode in pH 7.0 PBS by varying the concentrations of AA, DA, UA, Trp, and ${{\rm{NO}}}_{2}^{-}$. (**b**) Frontier molecular orbitals of AA, DA, UA, Trp, and ${{\rm{NO}}}_{2}^{-}$. (**c**) Schematic representation of the biosensing mechanism of the graphene/Ta wire electrode.

The individual electrochemical response of these analytes needs to be studied before the sensitivity of graphene/Ta wire electrodes toward the simultaneous detection of AA, DA, UA, Trp, and ${{\rm{NO}}}_{2}^{-}$ can be evaluated. The signal responses of graphene/Ta wire electrode for the oxidation of individual AA, DA, UA, Trp, and ${{\rm{NO}}}_{2}^{-}$ were investigated by differential pulse voltammetry (DPV) in 0.1 M PBS solution (pH 7.0) by varying their concentrations, as shown in Fig. 3a. The oxidation potentials from highest to lowest were ${{\rm{NO}}}_{2}^{-}$ (0.725 V) > Trp (0.58 V) > UA (0.25 V) > DA (0.115 V) > AA (−0.09 V), which shown in Fig. S2. The oxidation potential is generally governed by electron transfer from the analyte to the sensing electrode and hole transfer from the analyte to the counter electrode^49^. The graphene/Ta wire for use as a sensing electrode, the oxidation potential is dominated by both the difference in energy between the Fermi level of graphene (*E*_F_ ≈ −4.6 eV)^50^ and the highest occupied molecular orbital (HOMO) of analytes, and energy gaps between the HOMO and lowest unoccupied molecular orbital (LUMO) of the analytes. HOMO and LUMO energies approximately represent the a ability of molecules to donate and accept electrons^51^. H. Y. *et al*.^49^, reported that the oxidation potential increases with the increase of LUMO-HOMO energy gap. In this study, molecules and MO orbitals were prepared using GaussView 5.0 and all the calculations were performed using Gaussian V9.5. Moreover, all the molecules were fully optimized using the density functional theory (DFT)/B3LYP method with 6–31 G basis sets in water. The MO data was calculated at the B3LYP/6-311++G (d, p) level for AA and DA, while that of UA, Trp, and ${{\rm{NO}}}_{2}^{-}$ was at the RHF/AM1 level. Frontier molecular orbital analyses of the HOMO and the LUMO are summarized in Fig. 3b. The LUMO-HOMO energy gaps were, from highest to lowest, ${{\rm{NO}}}_{2}^{-}$ (11.19 eV) > Trp (8.6 eV) > UA (8.36 eV) > DA (5.55 eV) > AA (5.33 eV). The *E*_F_ (of graphene)-HOMO (of analyte) values decreases in the order UA (4.17) > ${{\rm{NO}}}_{2}^{-}$ (4.1) > Trp (4.04 eV) > AA (2.3) > DA (1.52). The effect of pH and solvent on the peak potential of the analyte is relatively large^52^. Dissociation constant (p*K*_a1_) values of AA, DA, UA, Trp, and ${{\rm{NO}}}_{2}^{-}$ are 4.17, 10.6, 5.75, 2.38 (p*I* = 5.89), and 3.29. The DA molecules did not dissociate into anions in pH 7.0 PBS, which further increased the oxidation potential of DA. Corresponding to the difference in the dipole moment, the HOMO and LUMO of Trp are localized on the indole ring, the LUMO of ${{\rm{NO}}}_{2}^{-}$ was highly asymmetrical, and the electron in the LUMO orbitals of Trp and ${{\rm{NO}}}_{2}^{-}$ was not delocalized onto the graphene layer, thus leading to more positive oxidation potentials of Trp and ${{\rm{NO}}}_{2}^{-}$ than those of AA, DA, and UA. Considering these factors, the order of electrocatalytic potentials was found to be NO_2_ˉ > UA > Trp > AA > DA.

The sensing mechanism of the graphene/Ta wire is shown in Fig. 3c. The anodic peaks correspond to the oxidation of hydroxyl groups to carbonyl groups of the furan ring in AA, oxidation of catechol to o-quinone in DA, oxidation of bridging double bonds to hydroxyl groups in UA, oxidation of the phenyl ring in Trp, and oxidation of the ${{\rm{NO}}}_{2}^{-}$ to ${{\rm{NO}}}_{3}^{-}$. The oxidation peak current is determined using the sum of the electron flow to the graphene and the hole flow to the Pt electrode. The oxidation current is affected by the adsorption strength between the graphene and the analytes. DA, UA, and Trp contain aromatic rings in their molecular structures (Fig. 3c). Graphene nanosheets have strong adsorption affinities to aromatic rings because strong π-π interactions between the free π-bonds of sp^2^ atoms from the six-benzene-ring graphene and the aromatic rings from to DA, UA, and Trp. Hence, high oxidation currents translate to higher sensitivity in the detection of DA, UA, and Trp than in the detection of AA and ${{\rm{NO}}}_{2}^{-}$.

Due to the availability of both large peak separations and enhanced currents, the graphene/Ta wire electrode is the best electrode for sensing the simultaneous detection of AA, DA, UA, Trp, and ${{\rm{NO}}}_{2}^{-}$. Freestanding graphene nanosheets are exposed to a lot of edges defects (Raman spectrum in Fig. 1f) and to the active sites from edge defects of graphene^53–55^.

### Determination of five species

Because pH has a profound effect on the electrochemical response of the sensing electrodes toward the simultaneous determination of AA, DA, UA, Trp, and ${{\rm{NO}}}_{2}^{-}$, the dependence of the peak potentials and currents of these five species oxidation on the different pH are shown in Fig. S3. In order to optimize sensitivity, selectivity, and practicability, pH 7.0 was selected for further experiments based on the results of the experiment shown in Fig. S3.

**Figure 4:**
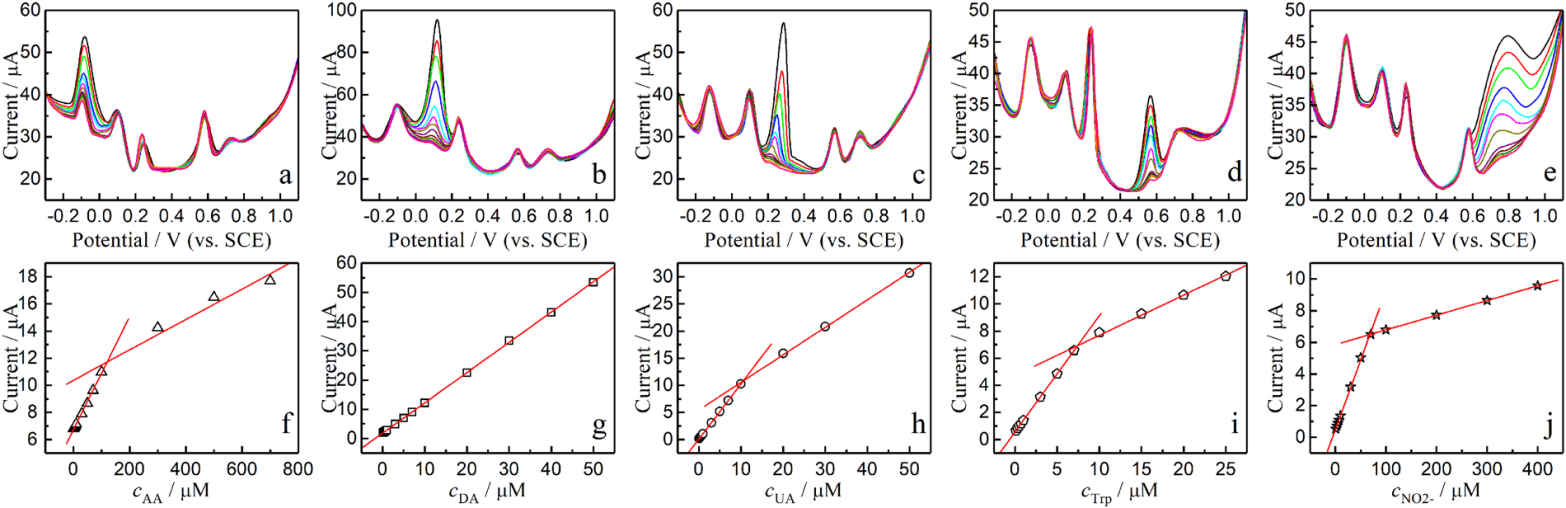
(**a**–**e**) DPVs of the graphene/Ta wire electrode for selective determination of AA, DA, UA, Trp, and ${{\rm{NO}}}_{2}^{-}$ in pH 7.0 PBS solution. (**f**–**j**) The plots of the oxidation peak currents versus variation in the concentrations.

**Figure 5:**
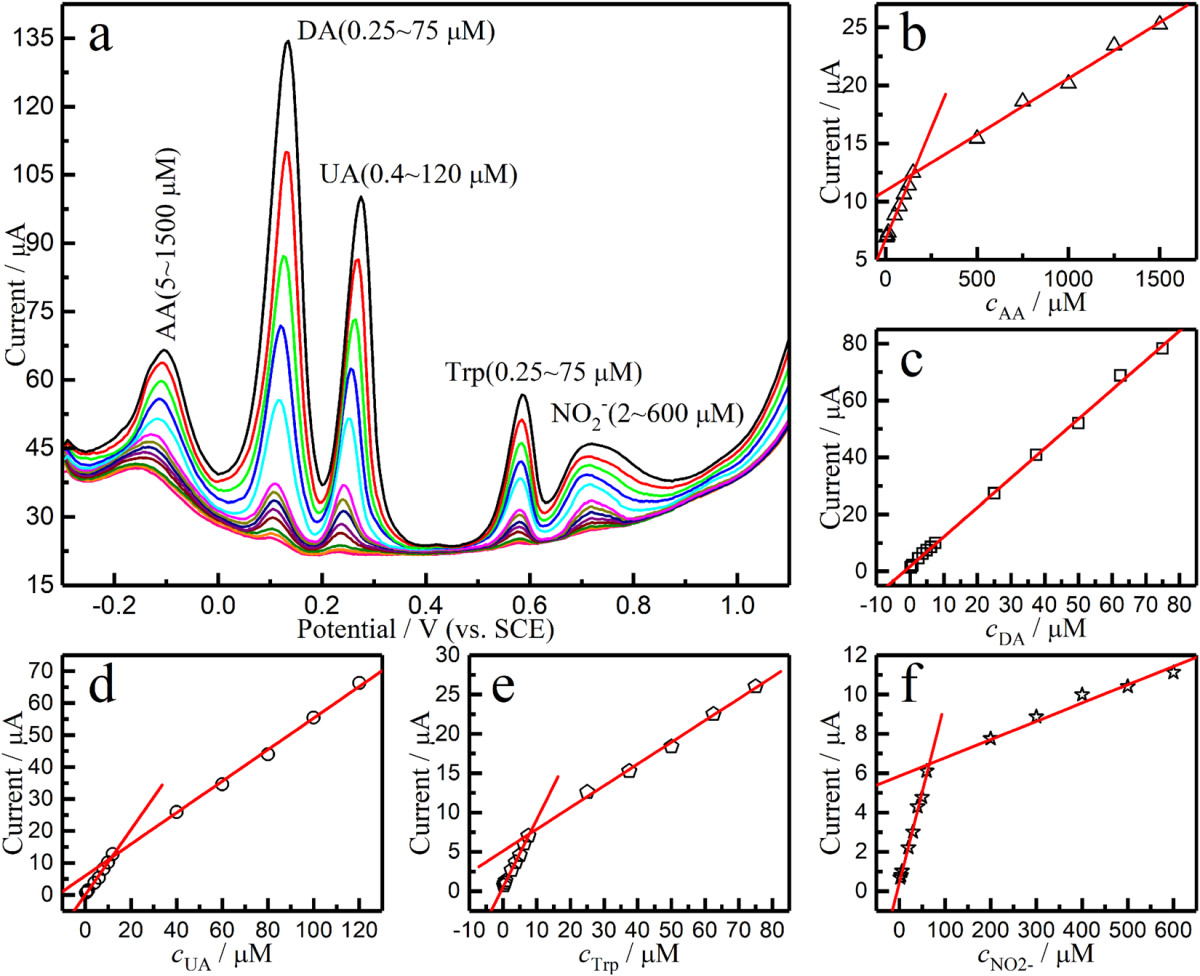
(**a**) DPV curves of graphene/Ta wire electrode in 0.1 M PBS (pH 7.0) with various concentrations of AA, DA, UA, Trp, and ${{\rm{NO}}}_{2}^{-}$. (**b**–**f**) Plots show peak currents versus the concentrations of AA, DA, UA, Trp, and ${{\rm{NO}}}_{2}^{-}$, respectively.

In order to identify the key parameters for detection of five species at graphene/Ta wire as a biosensor, the DPV curves of the mixtures of AA, DA, UA, Trp, and ${{\rm{NO}}}_{2}^{-}$ with various concentrations are measured selectively and simultaneously, as shown in Figs 4 and 5. Selective detection of five species showed the concentrations of four species to remain unchanged. Only one analyte showed any difference in concentration of one analyte. As shown in Figs 4 and 5, the peak currents were directly proportional to the concentrations of AA, DA, UA, Trp, and ${{\rm{NO}}}_{2}^{-}$. The concentration range, regression equation, and detection limit (LOD) are summarized in Table S1.

When the five analytes were detected simultaneously, i.e. when the concentrations of all five were changed, the calibration curves were linear and within the concentration ranges of 5–1500 μM for AA, 0.25–75 μM for DA, 0.4–120 μM for UA, 0.25–75 μM for Trp, and 2–600 μM for ${{\rm{NO}}}_{2}^{-}$. The slopes of the working curves changed at certain concentrations for AA, UA, Trp, and NO_2_ˉ. H. Y. *et al*. explained the phenomenon with the change of adsorption interaction^49^. The interface interaction of monolayer adsorption is strong, while that of multilayer adsorption is weak; hence, the slope of the working curve changed with increase in the concentration of analytes. Further, since the DA molecule contains an aromatic ring, a well-ordered organic layer was formed between the graphene and the aromatic ring of DA through π-π stacking interactions, which resulted in strong adsorption; therefor, the slope did not change within this concentration range. Compared with the flat graphene composite electrode, the sensitivity of the graphene/Ta wire electrode reached the same level, and the detection limits of DA, UA, and Trp are very advantageous^5, 26, 56^.

The LOD for AA, DA, UA, Trp, and ${{\rm{NO}}}_{2}^{-}$ was found to be 1.58, 0.06, 0.09, 0.10, and 6.45 μM, respectively. The lowest LODs obtained here for simultaneous determination of AA, DA, UA, Trp, and ${{\rm{NO}}}_{2}^{-}$ were comparable to or in some cases better than previously reported values. In Table S3, some of the analytical characteristics obtained in this work were compared to values previously reported in the literature^5, 21, 49, 56–59^. All this evidence supports the idea that the graphene/Ta wire electrode has an excellent ability to determine AA, DA, UA, Trp, and ${{\rm{NO}}}_{2}^{-}$.

The stability of the graphene/Ta wire electrode was also studied in this work. When the electrode was cyclically swept for 100 cycles, there was a 4.8% decrease in the initial response of the graphene electrode, indicating that the graphene electrode had excellent stability (Fig. S4). The reproducibility of the graphene electrode was determined using six different electrodes. The relative standard deviations (RSD) of the current responses for AA, DA, UA, Trp, and ${{\rm{NO}}}_{2}^{-}$ were 3.4%, 5%, 4%, 5%, and 4.5%, respectively.

Several forms of inorganic, organic, and biological interference were tested to determine whether they could affect the detection of AA, DA, UA, Trp, and ${{\rm{NO}}}_{2}^{-}$. These interferences were investigated at various concentrations in 0.1 M PBS (pH 7.0) containing 500 μM AA, 25 μM DA, 40 μM UA, 25 μM Trp, and 200 μM ${{\rm{NO}}}_{2}^{-}$ (Fig. S5). The results showed that concentrations of 500 μM CaCl_2_, 500 μM CuSO_4_, 500 μM KCl, 500 μM Mg(NO_3_)_2_, 500 μM Ni(NO_3_)_2_, 500 μM Zn(NO_3_)_2_, 500 μM Na_2_S, 500 μM FeSO_4_, 25 μM aniline, 25 μM phenol, 25 μM hydroquinone, 25 μM catechol, 25 μM resorcinol, 25 μM citric acid, 25 μM glucose, 25 μM L-alanine, 25 μM L-cysteine, 25 μM L-glycine, 25 μM L-lysine, and 25 μM L-tyrosine did not significantly influence the height of the peak currents. The tolerance limit was defined as the concentration giving an error of ≤5% in the determination of AA, DA, UA, Trp, and ${{\rm{NO}}}_{2}^{-}$ compounds. These results strongly demonstrate that the as-prepared graphene/Ta wire electrode performs has high selectivity and involves little interference.

### Real sample analysis

**Figure 6:**
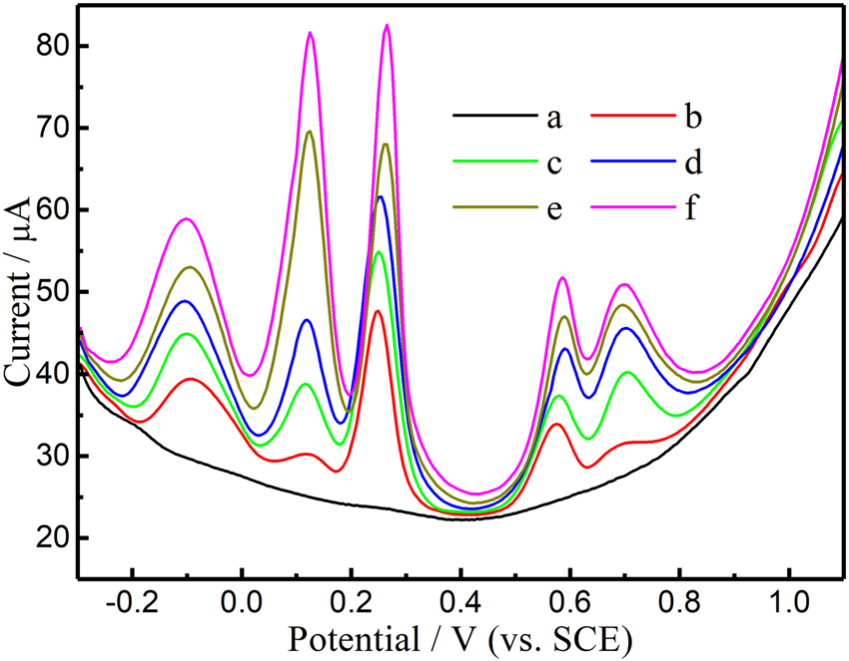
DPV curves of the graphene/Ta wire electrode in PBS the (a) absence and (b) presence of human serum and in (c–f) human serum containing additional AA, DA, UA, Trp, and ${{\rm{NO}}}_{2}^{-}$.

To evaluate the application of the graphene/Ta wire electrode, an attempt was made to detect five species in the human serum samples. Each sample was diluted 5 times with 0.1 M PBS (pH 7.0) before measurement. The electrochemical responses for detection of AA, DA, UA, Trp, and ${{\rm{NO}}}_{2}^{-}$ as shown in Fig. 6. The analytical results are presented in Table S4. The recovery was in the range of 94–104% for all analytes. We compared the results of various methods for the determination of concentration of the five analytes in serum (Table S5). In general, the concentrations of AA, DA, UA, and Trp are closer to those obtained by CL^60^, HPLC-CL^61^, GC/MS^62^, and UV methods^63^; however, the concentration of NO_2_ˉ is significantly higher than that measured by the spectrophotometric methods in the ref. 64. Since the sensitivities of various detection methods are different, and so are the serum samples, the results vary. However, the results show the presence these substances in human serum. These results indicated that graphene/Ta wire could produce results accurate enough for clinical diagnosis.

## Conclusions

Free-standing graphene nanosheets were here fabricated on Ta wire, which was used to selectively and simultaneously detect AA, DA, UA, Trp, and ${{\rm{NO}}}_{2}^{-}$ using a DPV method. The graphene electrode provided highly selective, sensitive results, with detection limits of AA, DA, UA, Trp, and ${{\rm{NO}}}_{2}^{-}$ of 1.58, 0.06, 0.09, 0.10, and 6.45 μM (S/N = 3), respectively. The key features of the graphene electrode responsible for its sensitivity improvement were found to be as follows: (i) free-standing nanosheets interlaced to form a network with mesoporous structures, which increased the surface area of the electrode, thereby facilitating ion adsorption on the surface of the electrode; (ii) high conductivity from CVD graphene nanosheets accelerated the ion diffusion and electron transfer. Free-standing graphene nanosheets are exposed to a lot of edges defects and to the active sites from edge defects of graphene. The Fermi level of graphene, HOMO, and LUMO of analytes affected the oxidation potential of the analytes. The two factors endowed the graphene electrode with high selectivity.

## Methods

### Reagents and materials

AA and DA were purchased from Aladdin Reagent Co., Ltd. (Shanghai, China). UA was purchased from Sigma Aldrich (U.S.). Trp and sodium nitrite (NaNO_2_) were purchased from Tianjin Guangfu Fine Chemical Research Institute (Tianjin, China). Phosphate buffered solutions (PBS) with various pH values were prepared using 0.1 M Na_2_HPO_4_ and 0.1 M NaH_2_PO_4_. Ultra-pure water was used in all experiments.

### Preparation of grapheme

Ta wires with a diameter of 0.6 mm were cut from 5 cm length wire-like substrates. The free-standing graphene nanosheets covered Ta wires, i.e. the graphene/Ta wire electrodes were prepared using a high-power direct current (DC) arc plasma jet CVD operating at gas recycling mode. The Ta wires were cleaned and attached to catalysts as follows before being placed in the deposition chamber: ultrasonic cleaning in ethanol and ultra-pure water, drying, immersion in the catalyst precursor solution (0.6 M Ni(NO_3_)_2_), and drying. The main deposition parameters are shown in Table S2.

### Characterization

The morphology of the CVD graphene obtained by scanning electron microscopy (SEM; Carl Zeiss MERLIN Compact, Germany), transmission electron microscopy (TEM; JEOL JEM-2100, Japan) and atomic force microscopy (AFM; Agilent 5500, U.S.) measurements, respectively. Sample treatment for AFM measurement: The graphene/Ta wire electrode was immersed in ethanol and sonicated in an ultrasonic bath for 48 h (600 W/40 kHz). During this time, a small amount of graphene sheet was peeled off and dispersed in ethanol. The graphene/ethanol solution was then sprayed onto the Si wafer and dried. The Raman spectrum of graphene was recorded on a Renishaw Raman microscope with an excitation laser wavelength at 532 nm. X-ray diffraction (XRD) spectra were created using a Rigaku Ultima IV x-ray diffractometer with CuK_α_ radiation (wavelength of 0.15406 nm).

The electrochemical performance was investigated using cyclic voltammetry (CV), differential pulse voltammetry (DPV), and electrochemical impedance spectroscopy (EIS) with a CHI 660E electrochemical workstation (Shanghai Chenhua Instrument Co., Ltd.). In a three-electrode system, a graphene/Ta wire electrode/or other sample electrodes, a platinum (Pt) sheet and a saturated calomel electrode (SCE) served as the working electrode, counter electrode, and reference electrode, respectively (Fig. 1a).

## Electronic supplementary material

Supplementary Information
