# Supplementary material for: CVD graphene as an electrochemical sensing platform for simultaneous detection of biomolecules
Source: Sci Rep. 2017 Aug 1;7:7044. doi: 10.1038/s41598-017-07646-2 (PMC5539141; doi:10.1038/s41598-017-07646-2)
Supplement: Supplementary file 1 — Supplementary Information [file 41598_2017_7646_MOESM1_ESM.doc]

**Supplementary Material**

**CVD graphene as an electrochemical sensing platform for simultaneous** **detection of biomolecules**

Xiaodan Wang^1^, Delan Gao^1^, Mingji Li^1,*^, Hongji Li^2,*^, Cuiping Li^1^, Xiaoguo Wu^1^, Baohe Yang^1^

^1^ *Tianjin Key Laboratory of Film Electronic and Communicate Devices, School of Electrical and Electronic Engineering, Tianjin University of Technology, Tianjin 300384, PR China*

^2^ *Tianjin Key Laboratory of Organic Solar Cells and Photochemical Conversion, School of Chemistry and Chemical Engineering, Tianjin University of Technology, Tianjin 300384, PR China*

*Corresponding Authors.

E-mail: limingji@163.com (M.J. Li); hongjili@yeah.net (H.J. Li).

Tel.: +86 022 60215346

**Table S1**

Parameters derived from DPV curves for individual, selective and simultaneous detection of AA, DA, UA, Trp, and NO_2_^-^.

| Method | | Selective | Simultaneous |
| --- | --- | --- | --- |
| Linear range  / µM | AA | 1-100; 100-700 | 5-150; 150-1500 |
|  | DA | 0.1-50 | 0.25-75 |
|  | UA | 0.1-10;10-50 | 0.4-12;12-120 |
|  | Trp | 0.1-7; 7-25 | 0.25-7.5; 7.5-75 |
|  | NO_2_^-^ | 1-70; 70-400 | 2-60; 60-600 |
| Linear equation  / µA, µM | AA | I=6.43+0.043 [AA]  I=9.25+0.013 [AA] | I=6.77+0.038 [AA]  I=10.96+0.010 [AA] |
|  | DA | I=1.32+1.177 [DA] | I=1.81+1.034 [DA] |
|  | UA | I=0.03+1.072 [UA]  I=4.83+0.525 [UA] | I=0.05+1.019 [UA]  I=6.03+0.493 [UA] |
|  | Trp | I=0.73+0.082 [Trp]  I=5.22+0.284 [Trp] | I=0.56+0.076 [Trp]  I=5.13+0.277 [Trp] |
|  | NO_2_^-^ | I=0.52+0.085 [NO_2_^-^]  I=5.14+0.009 [NO_2_^-^] | I=0.44+0.092 [NO_2_^-^]  I=5.86+0.009 [NO_2_^-^] |
| LOD  / µM | AA | 1.2 | 1.58 |
|  | DA | 0.04 | 0.06 |
|  | UA | 0.06 | 0.09 |
|  | Trp | 0.15 | 0.10 |
|  | NO_2_^-^ | 6.2 | 6.45 |

**Table S2**

Deposition parameters for the free-standing graphene nanosheets on Ta wire.

| Distance between the sample and the injection port (cm) | 1.5 |
| --- | --- |
| Base pressure (Pa) | 0.1 |
| Hydrogen flow rate (L min^-1^) | 2 |
| Ar flow rate (L min^-1^) | 1.5 |
| Chamber pressure (Pa) | 3000 |
| Pump pressure (Pa) | 13000 |
| Magnetron current (A) | 1.7 |
| Magnetron voltage (V) | 6.1 |
| Arc current (A) | 130 |
| Arc voltage (V) | 70 |
| Sample temperature (^o^C) | 1100 |
| Methane flow rate (L min^-1^) | 200 |
| Deposition time (min) | 15 |


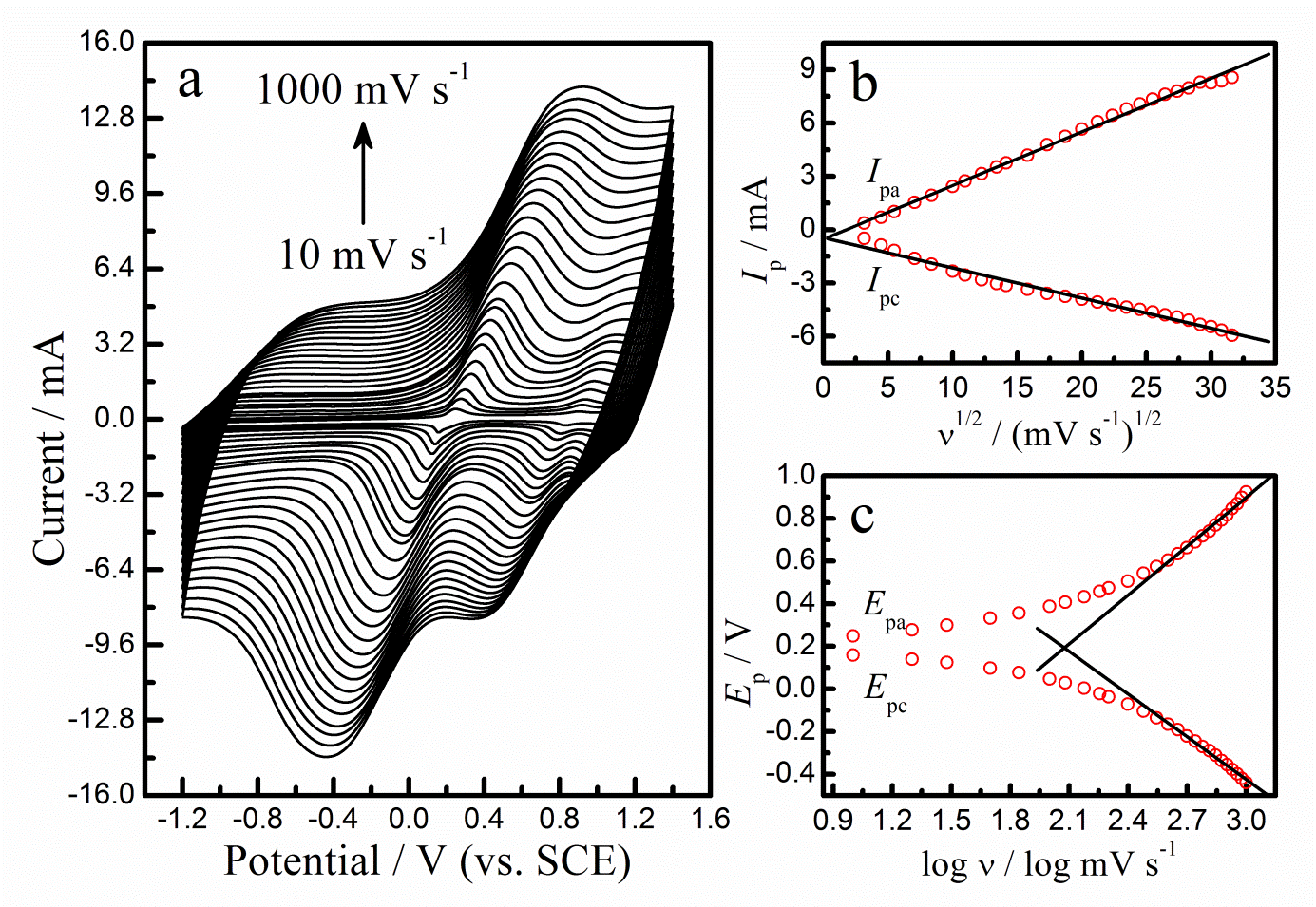


**Figure S1**. CVs of graphene/Ta wire electrode in 0.1 KCl solution in the presence of 5 mM K_3_Fe(CN)_6_ and 5 mM K_4_Fe(CN)_6_.

**
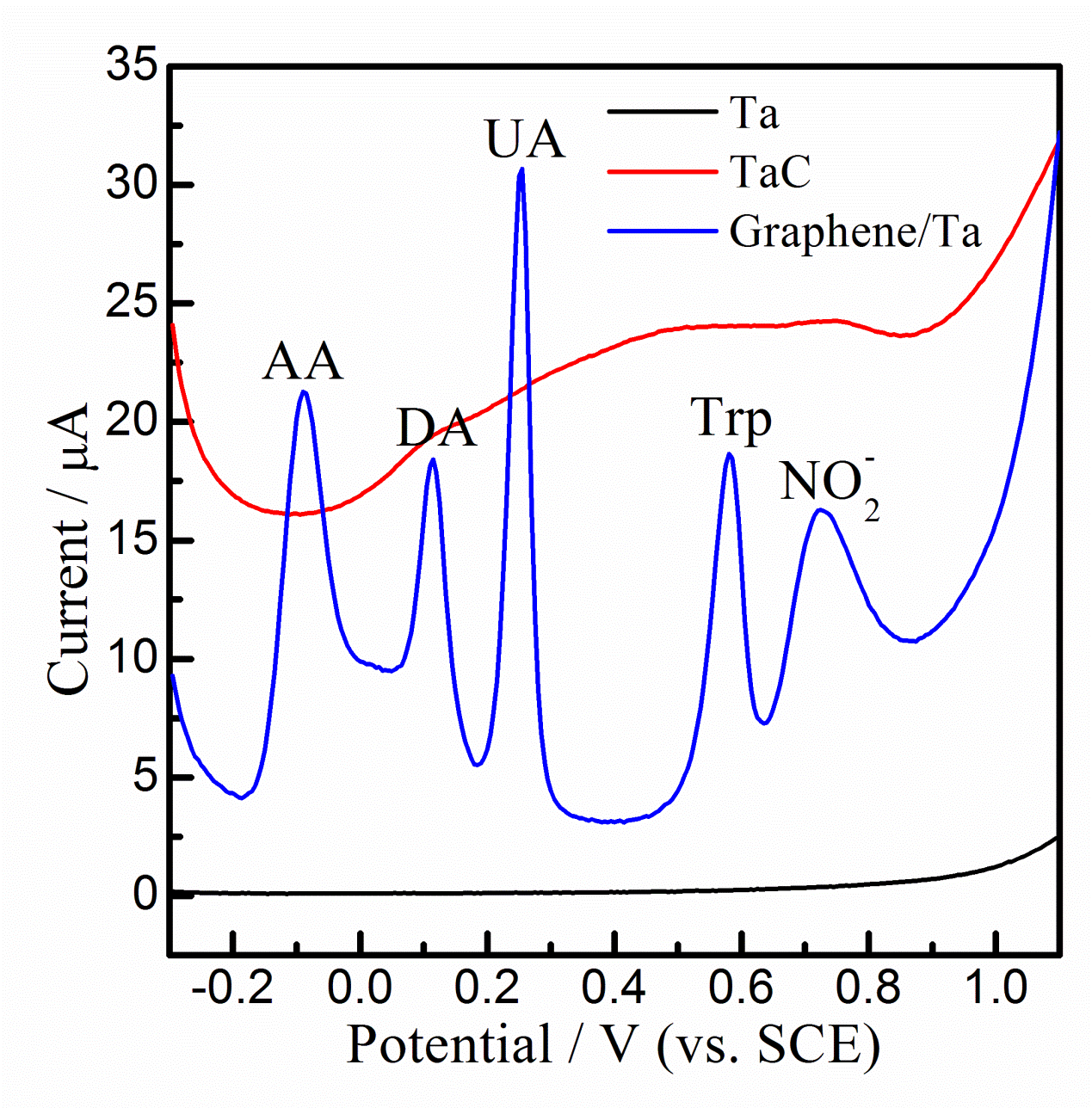
**

**Figure S2.** DPV curves of graphene/Ta wire, TaC/Ta wire, and Ta wire in pH 7.0 PBS containing 500 μM AA, 25 μM DA, 40 μM UA, 25 μM Trp, and 200 μM NO_2_^-^.

Fig. S2 shows the differential pulse voltammetry (DPV) curves of graphene/Ta wire, TaC/Ta wire, and Ta wire in pH 7.0 containing a mixture of 500 μM AA, 25 μM DA, 40 μM UA, 25 μM Trp, and 200 μM NO_2_^-^. Notably, five well-defined peaks were found at - 0.09, 0.115, 0.25, 0.58, and 0.725 V on graphene/Ta wire, the peak separations between AA and DA, DA and UA, UA and Trp, and Trp and NO_2_^-^ were 205, 135, 330, and 145 mV, respectively, indicating that the simultaneous determination of the five species was feasible. Their peak currents were much higher than those of other electrodes, which is enough to detect low concentrations of these electroactive compounds.


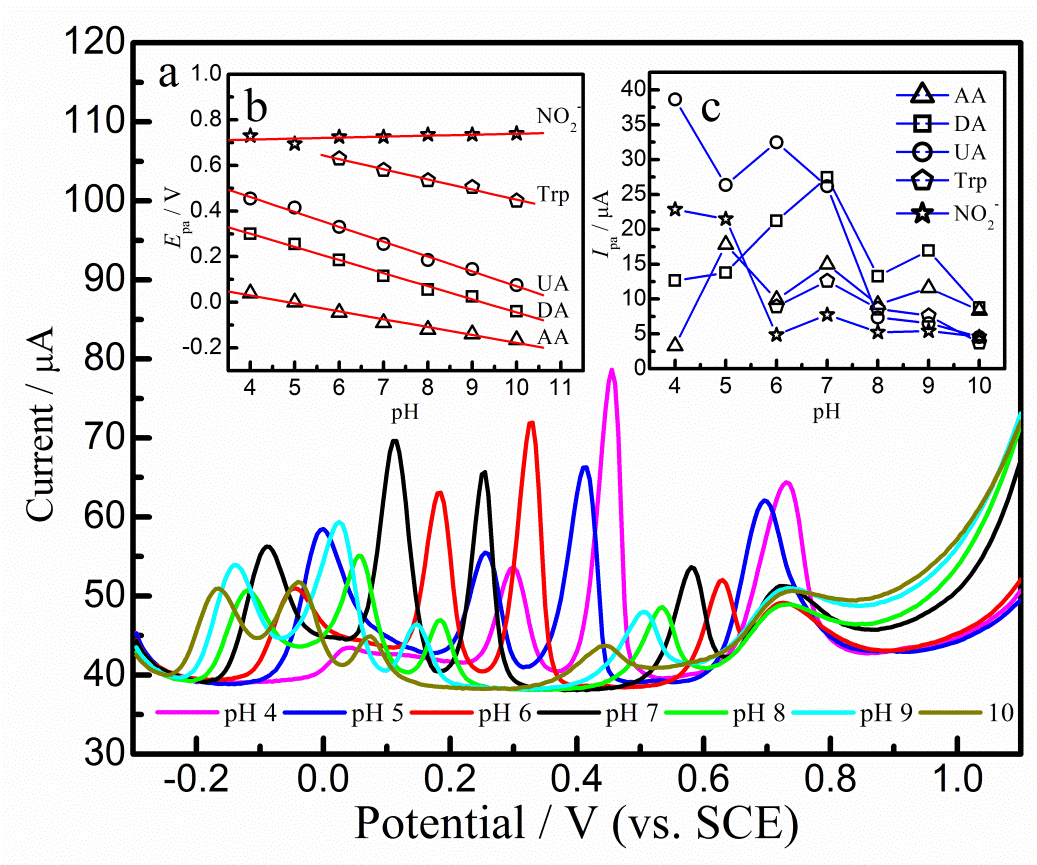


**Figure S3**. (a) DPVs recorded on graphene/Ta wire electrode in pH 4.0–pH 10.0 containing a mixture 500 μM AA, 25 μM DA, 40 μM UA, 25 μM Trp, and 200 μM NO_2_^-^. The effect of pH value on (b) the peak potentials and (c) the peak currents of the five species.

Because pH has a profound effect on the electrochemical response of the sensing electrodes toward the simultaneous determination of AA, DA, UA, Trp, and NO_2_^-^, the dependence of the peak potentials of these five species oxidation on the different pH are shown in **Fig. S3**. The peak potentials for the oxidation of AA, DA, UA, and Trp shifted to negative potential direction with increasing pH, which indicates that the protons participate in graphene/Ta wire electrode processes. The slopes obtained from the linear relationship between the applied potential and the pH for AA, DA, UA, and Trp were found to be 35, 58, 65, and 45 mV per pH (eqn (1)-(4)), which are similar to the anticipated Nernstian value (0.059 V per pH) for a two-electron/two-proton reaction. This suggests that electron uptake was accompanied by an equal number of protons.

*E*_p_ (AA)= -0.0346 pH + 0.168 R=0.990 (1)

*E*_p_ (DA)=-0.0575 pH +0.530 R=0.996 (2)

*E*_p_ (UA)=-0.065 pH +0.722 R=0.996 (3)

*E*_p_ (Trp)=-0.0445 pH +0.895 R=0.996 (4)

In addition, the dependence of the peak currents of AA, DA, UA, Trp, and NO_2_^-^ oxidation on the pH was investigated and the results are shown in Fig. S3c. The peak current of AA and DA initially increased with pH, reaching maximum values at about pH 5 and pH 6, respectively. The peak currents of UA and NO_2_^-^ decreased with pH. The peak current of Trp reached a maximum value at pH 7.0. In order to optimize sensitivity, selectivity, and practicability, pH 7.0 was selected for further experiments based on the results of the experiment shown in Fig. S3.

**Table S3**

Comparison some of the response characteristics of the different electrodes for the simultaneous determination of AA, DA, UA, Trp, and NO_2_^-^.

| Electrodes | pH | Delection limit (μM) | | | | | Method | Refs. |
| --- | --- | --- | --- | --- | --- | --- | --- | --- |
|  |  | AA | DA | UA | Trp | NO_2_^-^ |  |  |
| CuNPs/p-TAox/GCE | 4.0 | 5 | 0.03 | 0.16 | 0.16 | - | DPV,  Selectively | [^1^](#_ENREF_1) |
| CTAB-GO/MWCNT/GCE | 7.0 | 1 | 1.5 | 1 | - | 1.5 | DPV,  simultaneously | [^2^](#_ENREF_2) |
| Ag nanowire- rGO/SPCE | 7.4 | 0.81 | 0.26 | 0.3 | - | - | LSV,  simultaneously | [^3^](#_ENREF_3) |
| Nitrogen-doped carbon nanofiber/GCE | 4.5 | 50 | 0.5 | 1 | - | - | DPV,  simultaneously | [^4^](#_ENREF_4) |
| NiCo_2_O_4_/Nano-ZSM-5/GCE | 3.5 | 0.8 | 0.5 | 0.7 | 0.7 | - | DPV,  simultaneously | [^5^](#_ENREF_5) |
| ZnO nanowire array/graphene foam | 7.4 | 0.5 | 0.5 | 0.5 | - | - | DPV,  selectively | [^6^](#_ENREF_6) |
| Ag nanoparticles/rGO/GCE | 3.5 | 9.6 | 5.4 | 8.2 | 7.5 | - | DPV,  simultaneously | [^7^](#_ENREF_7) |
| Graphene/Ta wire | 7.0 | 1.58 | 0.06 | 0.09 | 0.1 | 6.45 | DPV,  simultaneously | This work |


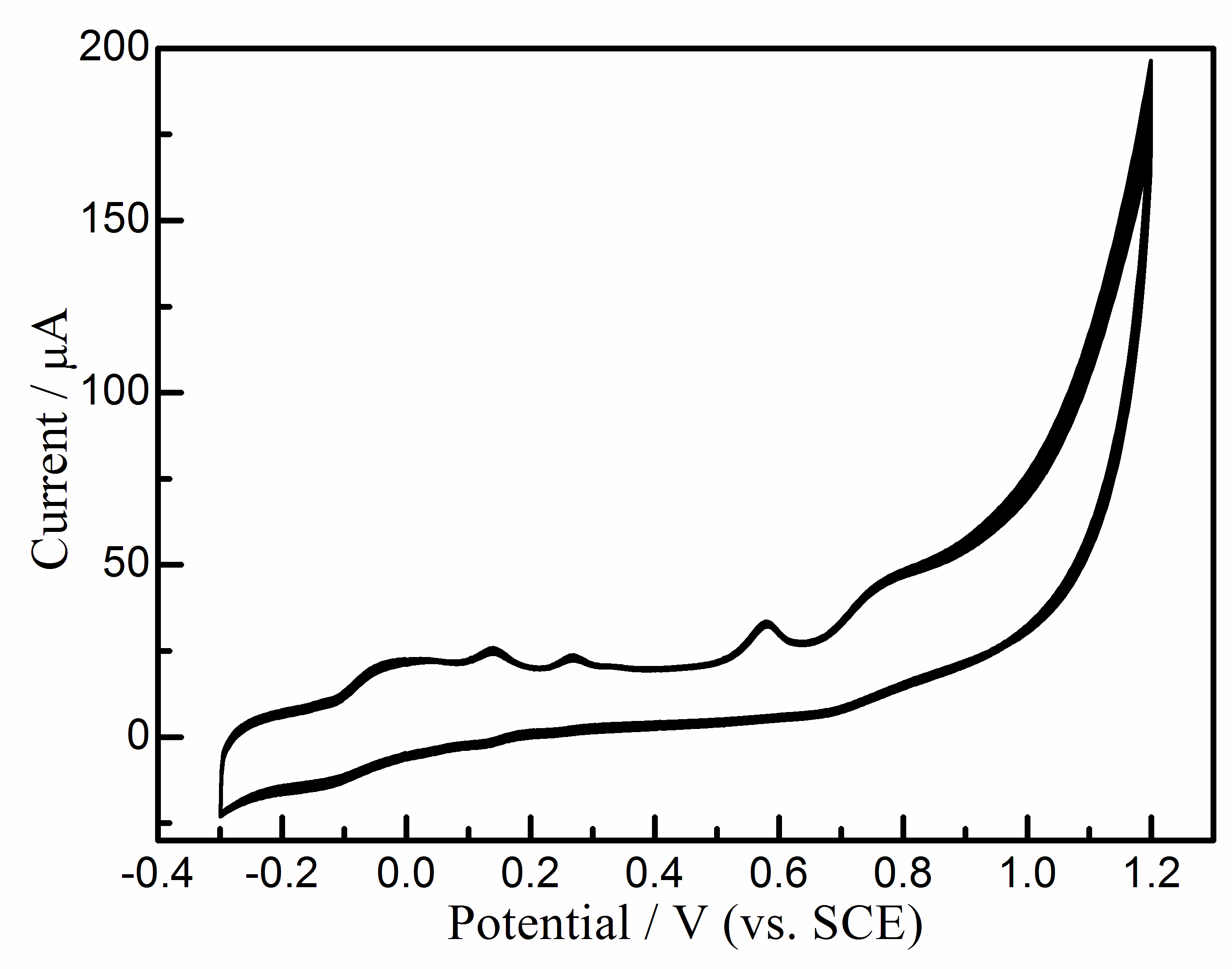


**Figure S4**. CV curves of graphene/Ta wire electrode in 0.1 M PBS solution containing a mixture 500 μM AA, 25 μM DA, 40 μM UA, 25 μM Trp, and 200 μM NO_2_^-^ for 100 continuous cycles.


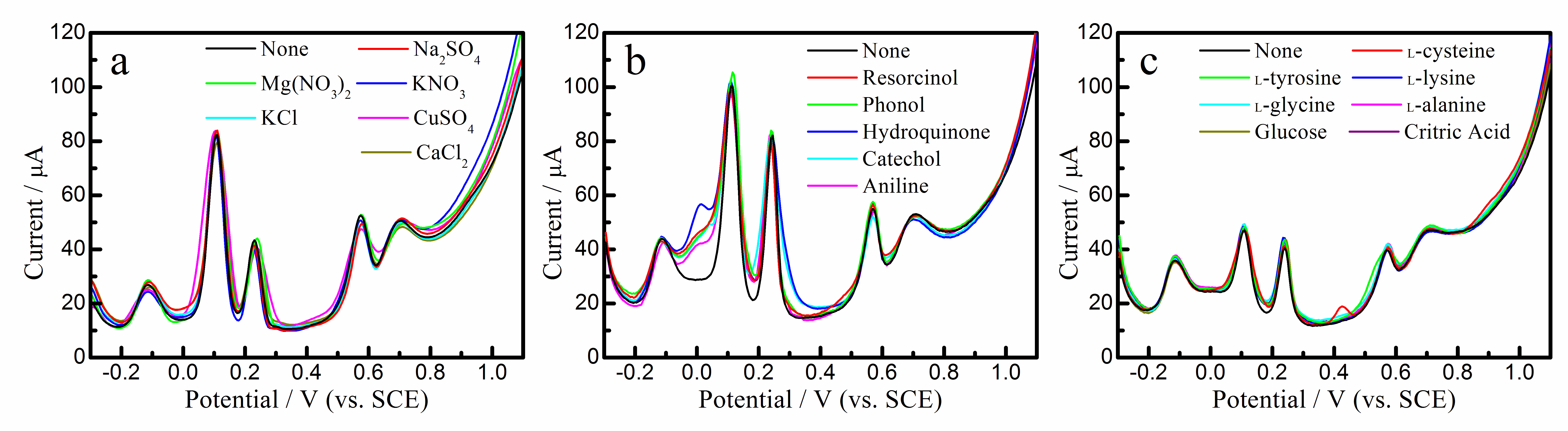


**Figure S5.** Interferences of some foreign substances for mixture of 300 μM AA, 20 μM DA, 50 μM UA, 20 μM Trp, and 300 μM NO_2_^-^. (a) The DPV curves of interferences citric acid, glucose, L-cysteine, L-glycine, L-lysine and L-tyrosine (each *c* = 20 μM) in presence of 300 μM AA, 20 μM DA, 50 μM UA, 20 μM Trp and 300 μM NO_2_^-^. (b) The DPV curves of interference from aniline, catechol, hydroquinone, phenol and resorcinol (each *c* = 20 μM) in the presence of 300 μM AA, 20 μM DA, 50 μM UA, 20 μM Trp and 300 μM NO_2_^-^. (c) DPV curves of interferences CaCl_2_, KNO_3_, Mg(NO_3_)_2_, Na_2_SO_4_, NaCl, and ZnCl_2_ (each *c* = 500 μM) in the presence of 300 μM AA, 20 μM DA, 50 μM UA, 20 μM Trp, and 300 μM NO_2_^-^.

**Table S4**

Determination of AA, DA, UA, Trp, and NO_2_^-^ in human serum samples (n=5)

| Smaples | | Added / µM | Found / µM | Recovery/% | RSD /% |
| --- | --- | --- | --- | --- | --- |
| 1 | AA | 0 | 12.32 | - | - |
|  | DA | 0 | 0.15 | - | - |
|  | UA | 0 | 31.51 | - | - |
|  | Trp | 0 | 7.63 | - | - |
|  | NO_2_^-^ | 0 | 7.63 | - | - |
| 2 | AA | 100 | 109.31 | 97.0 | 3.1 |
|  | DA | 6 | 6.37 | 103.7 | 2.3 |
|  | UA | 10 | 41.61 | 101.0 | 3.4 |
|  | Trp | 5 | 12.5 | 97.5 | 4.2 |
|  | NO_2_^-^ | 150 | 155.83 | 97.7 | 1.9 |
| 3 | AA | 300 | 302.30 | 96.7 | 2.1 |
|  | DA | 12 | 11.72 | 96.4 | 2.2 |
|  | UA | 20 | 51.26 | 98.8 | 2.1 |
|  | Trp | 10 | 17.9 | 103.1 | 3.7 |
|  | NO_2_^-^ | 250 | 245.86 | 94.6 | 3.2 |
| 4 | AA | 500 | 494.7 | 96.5 | 2.5 |
|  | DA | 30 | 31.14 | 103.3 | 3.6 |
|  | UA | 30 | 61.54 | 100.1 | 4.8 |
|  | Trp | 15 | 22.1 | 96.5 | 4.7 |
|  | NO_2_^-^ | 300 | 322.25 | 104.4 | 3.9 |
| 5 | AA | 700 | 737.56 | 103.6 | 3.1 |
|  | DA | 40 | 39.93 | 99.5 | 2.4 |
|  | UA | 55 | 87.07 | 101.0 | 3.5 |
|  | Trp | 25 | 33.57 | 103.7 | 3.1 |
|  | NO_2_^-^ | 400 | 399.79 | 97.6 | 4.7 |

**Table S5**

Comparison of different methods for the determination of AA, DA, UA, Trp, and NO_2_ˉ in human serum.

| Methods | Analytes in human serum | | | | | Refs. |
| --- | --- | --- | --- | --- | --- | --- |
|  | AA/ μM | DA/ μM | UA/ μM | Trp/ μM | NO_2_^-^/ μM |  |
| HPLC-FL | - | - | - | - | 0.25-0.54  (Rat) | [^8^](#_ENREF_8) |
| Spectrophotometric | - | - | - | - | 3.68±0.56 | [^9^](#_ENREF_9) |
| Capillary electrophoresis/UV |  |  | 321.22 |  | 6.6 | [^10^](#_ENREF_10) |
| CL | 14-32.20 | - | - | - | - | [^11^](#_ENREF_11) |
| HPLC-CL | - | 0.33 | - | - | - | [^12^](#_ENREF_12) |
| LC-MS | - | - | - | 86.13±4.12 | - | [^13^](#_ENREF_13) |
| LC-MS/MS |  |  |  | 57.4-63.6 |  | [^14^](#_ENREF_14) |
| GC/MS | - | - | 21.36-37.06 | - | - | [^15^](#_ENREF_15) |
| CL | - | - | 190-290 | - | - | [^16^](#_ENREF_16) |
| Graphene/Ta wire | 61.6 (12.32×5) | 0.75 (0.15×5) | 157.55  (31.51×5) | 38.15  (7.63×5) | 381.5  (7.63×5) | This work |

**References**

1. Wang, C. *et al.* Cu-nanoparticles incorporated overoxidized-poly(3-amino-5-mercapto-1,2,4-triazole) film modified electrode for the simultaneous determination of ascorbic acid, dopamine, uric acid and tryptophan. *J. Electroanal. Chem.* **741**, 36-41 (2015).

2. Yang, Y. J. & Li, W. K. CTAB functionalized graphene oxide/multiwalled carbon nanotube composite modified electrode for the simultaneous determination of ascorbic acid, dopamine, uric acid and nitrite. *Biosens. Bioelectron.* **56**, 300-306 (2014).

3. Li, S.-M. *et al.* Fabrication of a silver nanowire-reduced graphene oxide-based electrochemical biosensor and its enhanced sensitivity in the simultaneous determination of ascorbic acid, dopamine, and uric acid. *J. Mater. Chem. C* **3**, 9444-9453 (2015).

4. Sun, J. *et al.* Simultaneous determination of ascorbic acid, dopamine and uric acid at a nitrogen-doped carbon nanofiber modified electrode. *RSC Adv.* **5**, 11925-11932 (2015).

5. Kaur, B., Satpati, B. & Srivastava, R. Synthesis of NiCo_2_O_4_/Nano-ZSM-5 nanocomposite material with enhanced electrochemical properties for the simultaneous determination of ascorbic acid, dopamine, uric acid and tryptophan. *New J. Chem.* **39**, 1115-1124 (2015).

6. Yue, H. Y. *et al.* ZnO Nanowire Arrays on 3D Hierachical Graphene Foam: Biomarker Detection of Parkinson's Disease. *Acs Nano* **8**, 1639-1646 (2014).

7. Kaur, B., Pandiyan, T., Satpati, B. & Srivastava, R. Simultaneous and sensitive determination of ascorbic acid, dopamine, uric acid, and tryptophan with silver nanoparticles-decorated reduced graphene oxide modified electrode. *Colloids Surf., B* **111**, 97-106 (2013).

8. Shu-yu, Z., Qing, S., Li, L. & Xiao-hui, F. A simple and accurate method to determine nitrite and nitrate in serum based on high-performance liquid chromatography with fluorescence detection. *Biomed. Chromatogr.* **27**, 1547-1553 (2013).

9. Sastry, K. V. H., Moudgal, R. P., Mohan, J., Tyagi, J. S. & Rao, G. S. Spectrophotometric determination of serum nitrite and nitrate by copper-cadmium alloy. *Anal. Biochem.* **306**, 79-82 (2002).

10. Miyado, T. *et al.* Development of a novel running buffer for the simultaneous determination of nitrate and nitrite in human serum by capillary zone electrophoresis. *J Chromatogr A* **1014**, 197-202 (2003).

11. Chen, H., Li, R., Lin, L., Guo, G. & Lin, J.-M. Determination of L-ascorbic acid in human serum by chemiluminescence based on hydrogen peroxide-sodium hydrogen carbonate-CdSe/CdS quantum dots system. *Talanta* **81**, 1688-1696 (2010).

12. Chen, F.-N., Zhang, Y.-X. & Zhang, Z.-J. Simultaneous determination of epinephrine, noradrenaline and dopamine in human serum samples by high performance liquid chromatography with chemiluminescence detection. *Chin. J. Chem.* **25**, 942-946 (2007).

13. Ohashi, H. *et al.* Determination of l-tryptophan and l-kynurenine in Human Serum by using LC-MS after Derivatization with (R)-DBD-PyNCS. *Int. J. Tryptophan Res.* **6**, 9-14 (2013).

14. Takahashi, S. *et al.* Determination of l-tryptophan and l-kynurenine derivatized with (R)-4-(3-isothiocyanatopyrrolidin-1-yl)-7-(N,N-dimethylaminosulfonyl)-2, 1,3-benzoxadiazole by LC-MS/MS on a triazole-bonded column and their quantification in human serum. *Biomed. Chromatogr.* **30**, 1481-1486 (2016).

15. Sun, Y.-p., Chen, J., Qi, H.-y. & Shi, Y.-p. Graphitic carbon nitrides modified hollow fiber solid phase microextraction for extraction and determination of uric acid in urine and serum coupled with gas chromatography-mass spectrometry. *J. Chromatogr. B* **1004**, 53-59 (2015).

16. Amjadi, M., Manzoori, J. L. & Hallaj, T. Chemiluminescence of graphene quantum dots and its application to the determination of uric acid. *J. Lumin.* **153**, 73-78 (2014).
